# Simulations reveal variability in exposure to drier conditions during timing of budbreak for tree species of the mixedwood forests of Québec, Canada

**DOI:** 10.48130/forres-0024-0023

**Published:** 2024-08-07

**Authors:** Benjamin Marquis

**Affiliations:** Canadian Forest Service, Natural Resources Canada, Great Lakes Forestry Centre, 1219 Queen Street East Sault Ste. Marie, P6A 2E5, ON, Canada

**Keywords:** Climate change, Drought, Phenology, Temperature, Bud phenology models, Eastern Canada, SPEI

## Abstract

Due to climate change, the timing of budbreak is occurring earlier in temperate and boreal tree species. Since the warmer conditions also cause snow to melt earlier in the spring, the hypothesis that bud reactivation of tree species of the mixedwood forests of Québec would occur under drier conditions in the future and that species from the temperate forests with late budbreak would be most exposed to dry conditions was tested. The thermal-time bud phenology model was used to predict the timing of budbreak for early and late species using 300 and 500 growing degree-days as the threshold for the timing of budbreak. Climate data was obtained from four CMIP6 climate models from 1950−2100 for two socioeconomic pathways at two locations, one in the temperate forest and one in the boreal mixedwood forest. Using linear regressions, the anomaly, which results from the difference between the historical mean (1950−1980) and the yearly values in timing of budbreak was predicted by the anomaly in drought index (SPEI) per site, climate model, socioeconomic pathways, and species with early or late budbreak timing. Budbreak is expected to occur earlier in the future, whereas the temporal trends in SPEI remained weak during April and May. When paired with the anomalies in both timing of budbreak and drought index, analyses showed that budbreak could be expected to occur under drier conditions in the future. However, due to differences between climate models, it remains uncertain whether drought stress will begin earlier in the future.

## Introduction

Climate change is affecting tree phenology^[1]^. For many extra tropical tree species, the timing of meristem reactivation in spring occurs earlier whereas the timing of entrance into dormancy as well as the shedding of leaf in the fall occurs later^[2−6]^. In some species, however, budbreak or bud set can occur later and earlier in the spring and fall, respectively^[7,8]^. In addition, tree phenology models expect an increase in the variability of the predicted future budbreak timings due to climate change^[9,10]^. However, phenomenological observations of leaf phenology across elevation gradients in Europe suggests that tree species reactivate their meristems more uniformly under the ongoing warming^[11,12]^. In addition, Zani et al. suggested that the process of bud set and leaf shedding is linked with growth and reactivation of the meristem in spring and, would therefore occur earlier in the fall if budbreak and growth began earlier in the spring^[12]^. Therefore, even if empirical, experimental, model-based, or remote-sensing studies have been conducted at various spatial scales and on trees at different life stages, determining which leaf phenology model best fits different tree species is still a challenge^[13−15]^. These limits affect our ability to predict the timing (start and end) and length of the growing season. While the growing season is expected to last longer^[6,16]^, Usmani et al. found that bud set in black spruce (*Picea mariana* Mill B.S.P.), a common tree species in the boreal forest would occur even if air temperature remained above 20 °C^[16]^. Therefore, not all tree species may benefit from the expansion of warm weather in fall due to climate change. Even if the growing season length does not increase per se, its timings (start and end) would still be expected to change. As a result, leaf phenology may no longer be synchronized with the seasonal cycle of air temperature. For instance, earlier bud break and later entry into dormancy could increase exposure to freezing air temperatures in both spring and fall^[17,18]^, whereas earlier entry into dormancy when warm weather is still adequate for growth could limit carbon uptake, at least for some tree species.

It is important to determine whether this shift in leaf phenology and growing season length has a positive or negative impact on tree growth and survival to develop mitigation strategies and silvicultural practices that address the new environmental pressures that climate change is imposing on forest ecosystems. Many studies have focused on the impact of climate change on the intensity and severity of summer droughts^[19−21]^. However, it is unknown whether changes in leaf phenology can increase drought stress in spring and act together to increase drought severity in summer. Indeed, climate change is expected to increase the amount of precipitation that falls as rain rather than snow in winter, a meteorological phenomenon known as rain-on-snow (ROS) that can reduce snow cover by melting accumulated snow^[22−24]^. Moreover, warming air temperatures in winter and spring due to climate change could cause snow to melt earlier in spring^[24]^. If trees have not yet reactivated, water could runoff or have enough time to penetrate deeper into the soil than the depth at which the fine roots that absorb most of the water and nutrients are located^[25,26]^. Therefore, the decline in snow cover coupled with earlier melting could result in drier conditions in the spring and contribute to increasing drought intensity and durations throughout the growing season. Therefore, it is important to understand that climate change has larger impacts on the environment than just the increase in mean annual air temperature.

Northern temperate and boreal forests are subject to large seasonal fluctuations with minimum air temperatures well below freezing in winter, but also high air temperatures in summer^[27]^. Although the northern temperate and boreal forests are among the coldest forest biomes, dry conditions in summer can reduce tree growth and lead to fire, which is the most important natural disturbance in the boreal forest^[27−29]^. Therefore, in these two forest biomes, it is very important to determine whether the earlier onset of spring phenology coupled with earlier snowpack melting would increase spring drought. In this sense, Jing et al. suggested that spring leaf phenology is unlikely to converge between temperate and boreal forests under climate change, therefore, future spring drought exposure may vary between these two forest biomes^[10]^.

Due to the colder climate and the fact that more snow cover accumulates above the ground and the snow cover persists until later in the spring, budbreak occurs later in boreal forests than in temperate forests. It was then hypothesized that the impact of climate change on potentially drier conditions at the time of budbreak in spring is less likely in the boreal forest than in the northern temperature forest. Using the thermal time bud phenology model, the potential future timing of budbreak was determined for early and late reactivating species based on growing degree-day thresholds of 300 for early and 500 for late bursting species based on the literature^[30]^. The standardized precipitation evapotranspiration index (SPEI) was then calculated and it was determined whether the anomaly in the timing of budbreak increased as the anomaly in SPEI increased in the month of budbreak at two sites, one in the South (northern temperate forest) and one in the North (boreal mixedwood forest) in Québec, Canada.

## Materials and methods

### Study area

**Figure 1:**
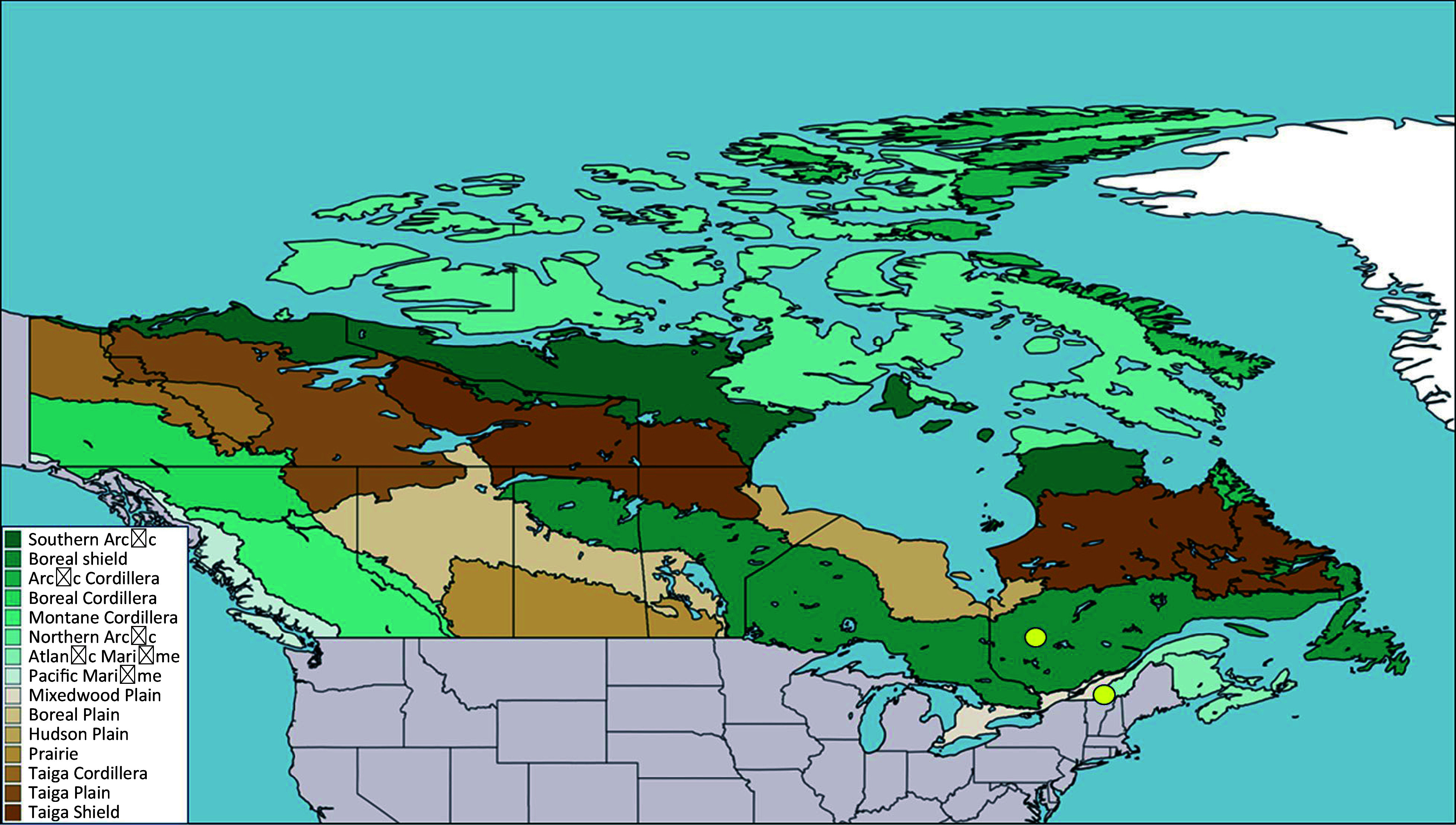
Map showing the locations (yellow dots) of the two study sites in Québec, Canada. The colored area represents the vegetation zones of Canada according to the Canadian National Vegetation Classification.

This study was set in two sites representative of two ecologically and economically important forest types in Québec, a southern site in the northern temperate forest and a northern site in the boreal mixedwood forest. The northern temperate forest (45.33°N, −72.22°W) is composed primarily of mature broadleaves tree species such as maples and birches and a few conifer species, while the boreal mixedwood forest (49.0°N, −77.00°W) is primarily composed of conifer species such as spruces and fir and a few broadleaves species such as birches, red maple, and aspen (Fig. 1)^[31,32]^. These sites cover a climate gradient and therefore allow the analysis of the risk of increased drought in spring due to climate change. For instance, at the northern site, the climate normals for the period 1981−2010 show that the mean annual air temperature is colder by 4.6 °C, being 1 °C (Lebel sur Quevillon weather station, station ID 7094275), compared to 5.6 °C at the southern site (Magog weather station, climate ID 7024440). However, the seasonal extremes in air temperature can reach similar values, reaching −43 °C at the northern site and −38 °C at the southern site during the winter and 34.4 °C at both sites during the summer. Moreover, the northern site is not as wet as the southern site, receiving a total sum of precipitation of 928 mm instead of 1,142 mm but has deeper snow cover in April reaching 16 cm instead of 2 cm at the southern site. Therefore, the high summer temperatures coupled with less precipitation in the northern site, may result in drier summer conditions at the northern site even though it is colder on average. However, increased snowpack and colder air temperatures in the northern site could limit spring droughts.

### Phenology model

The thermal time model was used to simulate the timing of budbreak during the period 1950−2100. This model considers that budbreak occurs once trees have accumulated enough heat in spring^[33]^. Heat is represented in the form of growing degree-days, which is the sum of the average daily temperature above 0 °C^[34]^. The summation started on January 1^st^ of each year. Timing of budbreak was determined as the day of the year (DOY) a specific growing-degree day threshold was reached, which was set at 300 and 500 to represent both early and late bursting species^[30]^. These two thresholds were based according to commonly used thresholds in the literature^[35−42]^. The thermal time model was used because at both sites, cold temperature allowing for chilling completion is expected to occur even under climate change, hence, the importance of the interplay between chilling and forcing is reduced^[30]^. Since long-term phenology records are scarce in Québec compared to Europe or Asia where long-term tree phenology records of budbreak exists (www.pep725.eu^[2]^), testing and calibrating model parameters of many phenology models based on past observations is not as feasible^[15,43]^, hence a commonly used and simple phenology model was used for which parameter values exist in the literature.

### Climate data

The maximum and minimum daily air temperature as well as the daily precipitation sum for the period 1950−2100 were retrieved from four chains of global and regional climate models (ACCESS-CM2, CanESM5, MIROC6 and GFDL-ESM4) that are available on the Pacific Climate Impacts Consortium website for two different socioeconomic pathways (SSP 2-4.5 and SSP 5-8.5) (https://data.pacificclimate.org/portal/downscaled_cmip6/map/) at both locations. These chains of global and regional climate models are set at a resolution of 10 km and the downscaling followed the bias-corrected constructed analogs and the Quantile Delta Mapping^[44,45]^.

Of course, many other climate models are available, for instance, the PAVICS website provides climate data from 26 climate models. Not all models have the same warming trend or the same ability at simulating past climate, indeed CanESM5 is expected to be warmer than the other three climate models tested^[46]^. Testing models with different warming trends allow determining the variation in potential exposure to future drought during budbreak timing.

Using the Hargreaves potential evapotranspiration calculated with the PET function and the SPEI function from the SPEI library^[47]^ available in the R software for Statistical Computing version 4.3.1.^[48]^ The monthly standardized precipitation evapotranspiration index (SPEI) per year, climate model, and site were calculated. This drought index is built so that negative values represent dry conditions, positive values represent wet conditions, and values close to zero represent normal conditions^[49]^.

### Statistical analyses

#### Past change in standardized potential evapotranspiration index at the timings of budbreak

To determine if the timing of budbreak is projected to advance under climate change, simple linear regression analysis was used, having the day of the year (DOY) on which budbreak was expected to occur as the response variable and the years, the climate models, the sites, the growing degree-day threshold (300 and 500) used to differentiate early from late busting species and the socioeconomic pathways as well as interactions between each pair of variables as predictors. The best set of predictor variables was identified following stepwise backward variable selection. The stepwise backward variable selection method was also used to identify the best set of variables (years, climate model, sites, socioeconomic pathway and the interactions between each pair of variables) predicting the temporal trends in monthly SPEI.

To determine if the change in timing of budbreak could affect exposure to dry conditions, the month in which the timing budbreak was predicted to occur between 1950−1980 was first identified since it represents the historical timing of budbreak. Second, the average of the drought index during this historical timing of budbreak was calculated, which represents the baseline exposure to drought during the timing of budbreak. Third, the difference between the historical drought index during the budbreak period with the drought index value during the month of budbreak predicted by the phenology model for each climate model and site was calculated. This difference represents the anomaly in the drought index during the timing of budbreak. To determine if budbreak is expected to occur under drier conditions compared to the past, simple linear regression having the anomaly in budbreak timing as response variables and the anomaly in drought index per site, climate models, socioeconomic pathways, the year, and a factor variable differentiating between species with early or late timing of budbreak as well as the interactions between each pair of predictors were used. All analyses were conducted in R software for Statistical Computing version 4.3.1^[48]^.

## Results

### Temporal trends in simulated timing of budbreak

**Figure 2:**
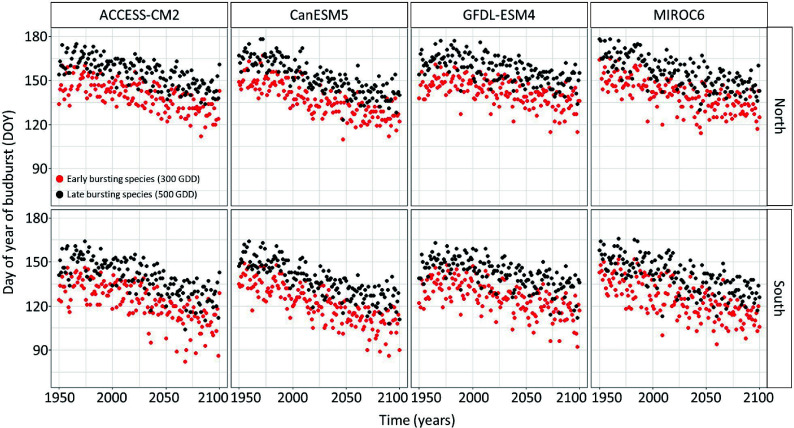
Timing of budbreak predicted by the thermal time bud phenology model, which considers budbreak to occur once 300 and 500 growing degree-days have accumulated under the SSP-2.45 for species showing early and late budbreak respectively, per site and chains of global and regional climate models. Red dots represent species with an early timing of budbreak whereas black dots represent species with a late timing of budbreak.

**Figure 3:**
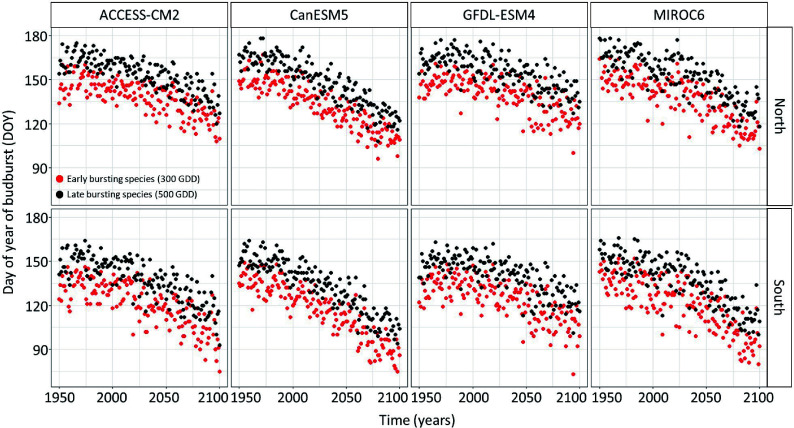
Timing of budbreak predicted by the thermal time bud phenology model, which considers budbreak to occur once 300 and 500 growing degree-days have accumulated under the SSP-5.85 for species showing early and late budbreak respectively, per site and chains of global and regional climate models. Red dots represent species with an early timing of budbreak whereas black dots represent species with a late timing of budbreak.

The linear regression model predicting the timing of budbreak under climate change showed an advance in the timing of budbreak in the future (Figs 2 & 3). This advance is expected to occur at a faster rate in the South since the interaction between site and year was statistically significant (Supplemental Table S1) and under the extreme socio-economic pathway SSP-5.85 since the interaction between the socioeconomic pathway and the year were statistically significant (Supplemental Table S1). The simulated timing of budbreak also varied per chains of global and regional climate models (Supplemental Table S1); indeed, the climate model CanESM5 predicts the most advanced timing of budbreak whereas the climate model GFDL-ESM4 predicts the least advanced timing of budbreak. The timing of budbreak simulated using the thermal time model seems to follow a linear trend since the linear regression explains 78% of the variation in the simulated timing budbreak.

### Temporal trend in SPEI

**Figure 4:**
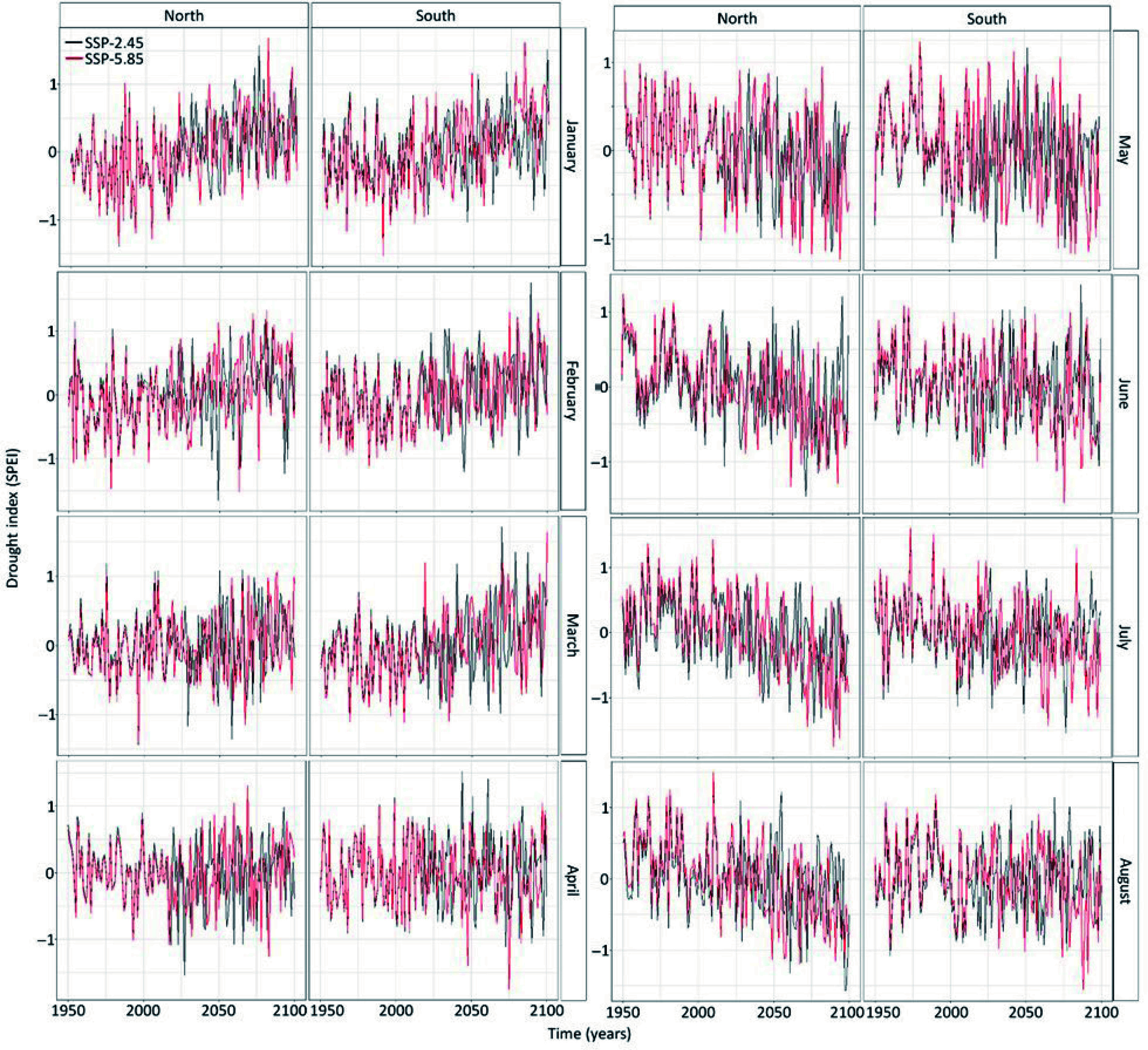
Temporal trends in the monthly drought index (SPEI) from January to August at both sites calculated from the four climate models. The black line shows the predictions of the drought index under the socioeconomic pathway 2.45 whereas the red line shows the predictions of drought index under the socioeconomic pathway 5.85.

The drought index showed important variations in its temporal trends between months. For instance, during the winter (January, February, March) the drought index is expected to be more positive, hence these months are expected to be wetter in the future (Fig. 4). The temporal trend in the drought index is near zero in April and turns negative during the months of spring and summer (Fig. 4). Hence spring and summer are expected to be drier in the future. The drought stress is also expected to increase at the northern site (Fig. 4). Surprisingly, there was no difference in the drought index between climate models and the difference in the drought index per socioeconomic pathways were marginal since *p*-values were below 0.1 but above 0.05 (Supplemental Table S2). While these relations were statistically significant, the linear regression only explains 3% of the variation in the data, indicating high inter-annual variability in drought.

### Temporal trends in drought index during the timing of budbreak

**Figure 5:**
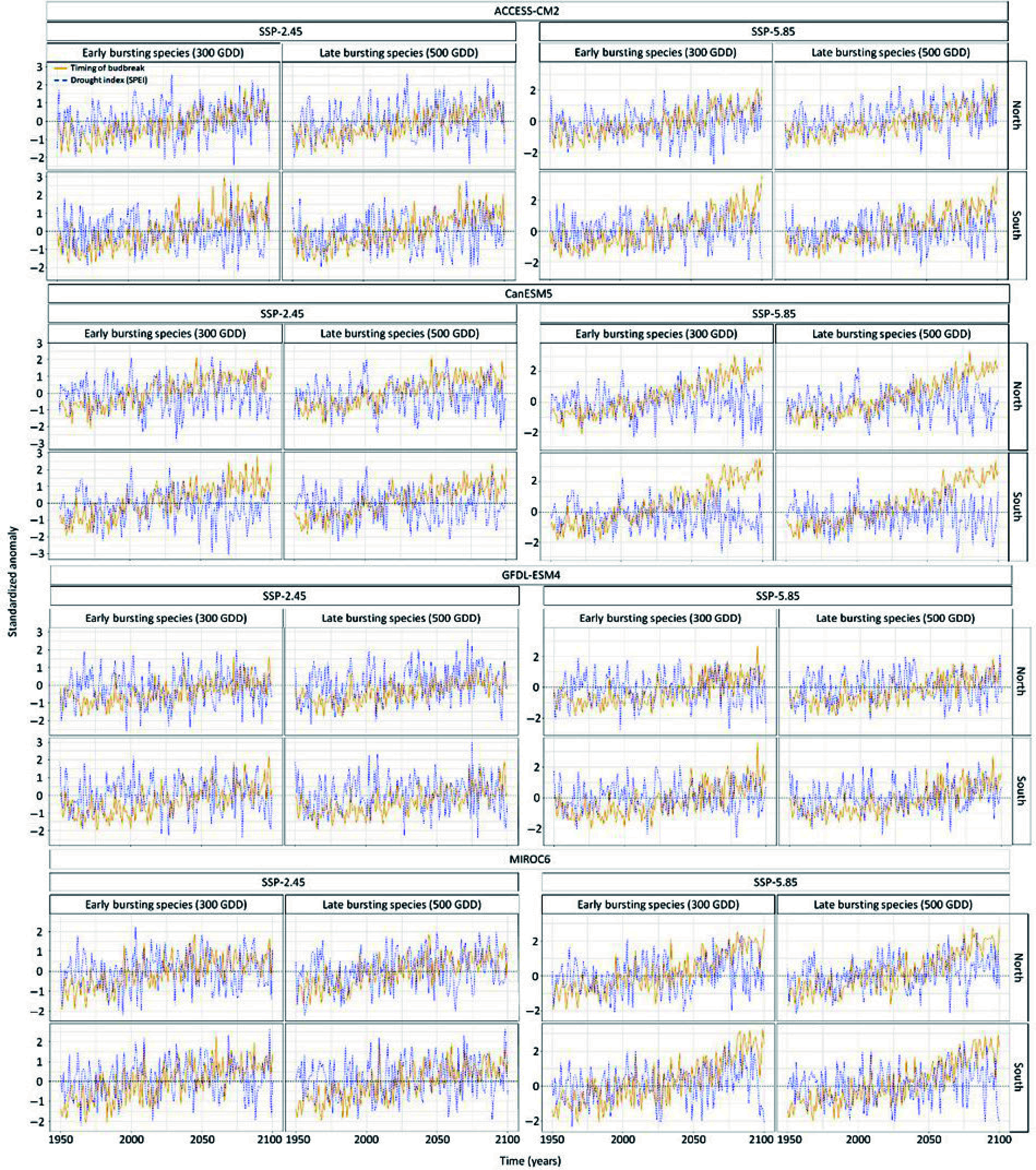
Standardized temporal trends in the anomaly of species exhibiting early and late timing of budbreak and in anomaly in drought index per climate model, socioeconomic pathways, sites. Standardization was conducted by subtracting each value by the mean and then divide it by the standard deviation. This statistical process was done using the scale function in the R software for statistical computing. The blue line shows the scaled anomalies in the drought index whereas the orange line shows the scaled timing of budbreak.

**Figure 6:**
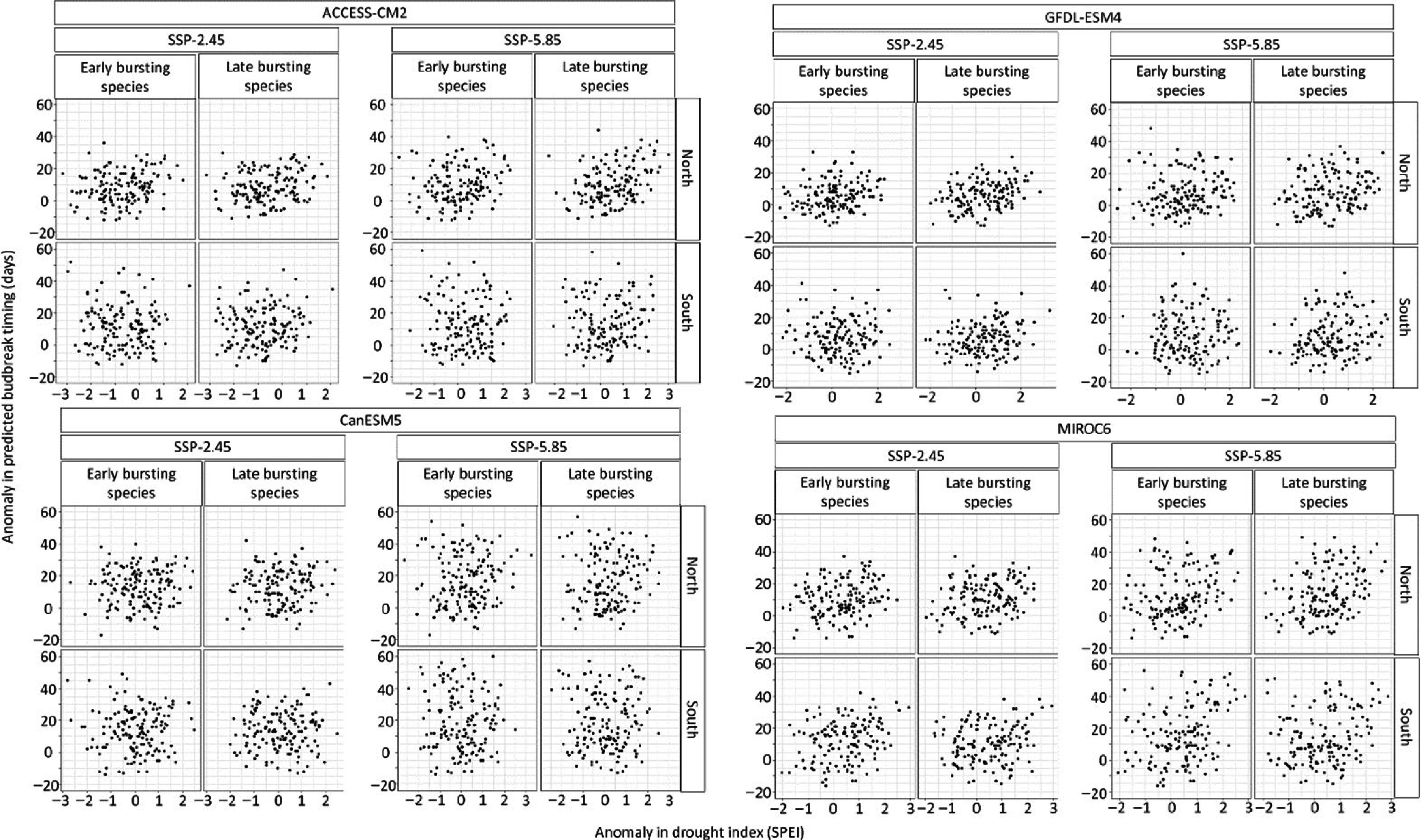
Relation between the anomaly in species exhibiting early (once 300 growing degree-days have accumulated) and late (once 500 growing degree days have accumulated) timing of budbreak and the anomaly in drought index during timing of budbreak per site, climate model, and socioeconomic pathways.

The temporal trend in the drought anomalies during the timing of budbreak varied per climate model, being stronger for the climate model MIROC6 and GFDL-ESM4 compared to CanESM5 and ACCESS-CM2 (Figs 5 & 6). Hence some climate models expect budbreak under drier conditions whereas other climate models do not expect such a result. Moreover, the timing of budbreak under dry conditions is most expected at the northern site (Figs 5 & 6). Still, drier conditions at the southern site could occur since the interaction between the years and the site is positive and significant (Supplemental Table S3). While the anomalies in the timing of budbreak increased under the extreme socioeconomic pathway SSP-5.85 compared to SSP-2.45, the drought index did not vary enough between socioeconomic pathways to have significant interactions between the anomalies in the drought index and the socioeconomic pathways. Results also show that species with early and late timing of budbreak have similar exposure to dry conditions, however, this finding was likely due to the setting of the drought index at a monthly scale. Indeed, in the South, species with early and late budbreak were predicted to occur during May under both socioeconomic pathways. Timing of budbreak was only expected in June at the northern site for species with late timing of budbreak. This linear regression model explained 64% of the variation in the anomaly of the simulated timing of budbreak.

## Discussion

In this study, the hypothesis that under warmer climates the breaking of buds in spring could occur under the drier conditions and advance the risk of drought stress in two forest types in eastern Canada was tested. By selecting two sites in different forest types found along a South to North gradient and comparing species with early and late bud burst timing, it was determined where and for which species, based on their budbreak strategy (early vs late), the advance in budbreak timing under the warmer climate could lead to greater drought stress. This study is acknowledged to be a simulation exercise and would require field observation and experimental testing, but it provides an initial estimate of the potential increase in dry conditions during budbreak timing.

As expected, the simulations of the timing of budbreak by the thermal-time model shows an earlier timing of budbreak due to climate change. Even if other phenological models could be used, the difference in their predictions of the timing of budbreak is of only a few days^[44,50]^, hence the variability between phenological models may not be too important considering that drought was measured on the monthly scale. Therefore, the results suggest that budbreak timing may occur under drier conditions in the future is likely irrespective of the bud phenology model used but is highly dependent on the climate model that is used since climate models differ in their predictions of exposure to drought during the period of budbreak. Therefore, the present hypothesis that the timing of budbreak would occur under drier conditions is only partly supported since it varied per climate model and because of the high inter-annual variability in drought index. While the hypothesis remains open and further empirical testing is required, it is unlikely that budbreak will occur under wetter conditions, therefore the possibility that the beginning of drought stress will occur earlier needs to be considered. According to the remote sensing study conducted by Buermann et al. over the temperate and boreal forests of Canada, when budbreak occurs earlier, the gain in productivity induced by this earlier start is canceled by the increase in drought during the summer^[51]^. If drought stress starts earlier in spring, it could have an additive effect when coupled with summer drought and lead to a drastic negative impact on trees. In addition, the present results lead to the more general conclusion that bud phenological models are likely to be more sensitive to climate data input than the various ways in which bud phenology models can be expressed. The fact that the biological interpretation of drought during the timing of budbreak varies when using different climate models call for great care when interpreting tree response under climate change. Moreover, using a single climate model or the average of many climate models can reduce the simulated impact climate extremes can have on trees. Using many climate models when determining tree response to climate change should become a standard practice as was carried out by Marquis & Lajoie^[52]^.

Contrary to the present hypothesis, species with a late budbreak strategy did not necessarily show an increase in relative drought exposure at the time of budbreak compared to species that burst buds earlier. It is acknowledged that the late reactivating tree species can be more exposed to drought since snow has had more time to melt and water has been able to penetrate deeper into the soil, and therefore are less accessible to tree species with shallow root systems. However, the relative difference scaled using the historical timing of budbreak per budbreak strategy did not increase for species breaking their buds late. Therefore, even if more snow accumulates in the boreal forest than in the northern temperate forest, the timing of budbreak at the northern site is expected to occur under relative drier conditions compared to trees at the southern site. It has been suggested that tree phenology can interact with drought recovery and that drought stress may increase at the time of budbreak in trees growing in the boreal mixedwood forest, as this forest type has a harder time to recover from droughts than in the northern temperate forest^[53]^. Since tree species differ in their growth rate, with some trees growing faster in spring whereas others grow more during summer^[53,54]^, forest management such as assisted migration or assisted gene flow could try to identify which tree species burst early and grows more in spring since it might limit the drought stress during the period they grow the most^[55]^. However, other environmental stressors such as the risk of late frost must be considered since early reactivating species can be more exposed to late frost^[18,56]^. Hence, trying to limit spring drought but increasing the risk of late frost will not necessarily optimize the growth performance of trees. An integrated assessment of both late frost and drought during spring and summer need to be conducted to determine how best to manage forest stands under climate change^[57,58]^.

An important consideration when pairing drought stress and timing of budbreak is the scale at which both phenomena occur. Timing of budbreak is at the daily scale whereas drought index is often aggregated at the yearly, seasonal (mostly summer) or at the monthly scale, which seems to mismatch. However, the timing of budbreak results from complex physiological mechanisms that occur prior the timing of budbreak that require the rehydration of the tree^[34,59]^. In addition, the budbreak process from buds swelling to leaf fully open takes about 20 to 30 d^[14,39,60]^. Hence, comparing the timing of budbreak with the monthly drought index seems biologically relevant. Future research should empirically test these arguments by recording tree phenology and quantifying xylogenesis using dendrometers and microcores to monitor cambial activity. The important limitation of such a comparison is that the drought index is based on fixed calendar dates to calculate the monthly drought index, however, budbreak could overlap between two months, hence, developing a more flexible drought index that could be calculated over 30 d but with a moving starting start date might provide a better assessment of the dry conditions during meristem reactivation and rehydration. This moving starting day would also be helpful at determining exposure to drought for species showing early and late timing of budbreak since, in this study, if budbreak occurs during the same month for both early and late bursting species, they would share the same anomaly in the drought index. Hence, it remains hard to assess the differences in drought stress at the time of bud break between early and late bursting species. Novel research projects should aim to empirically quantify the temporal shifts in the relationships between tree growth and drought (SPEI) between early and late bursting species using dendroecology in both forest ecosystems.

Another limitation of this study is the use of daily precipitation without distinguishing the different phases of water (snow vs rain), which limits the conclusion about the effects of rain-on-snow on the snow cover. If rain-on-snow does indeed melt snow cover and reduces the amount of water available to trees during spring the hypothesis that the timing of budbreak would occur under greater drought intensity could be better assessed than using a simple precipitation value. Hence, climate organizations and climate data providers should, when possible, provide the distinctions in the different phases of water and on snow cover. This could improve not only predicting when drought stress would start but could also help in identifying the start of fire-prone conditions and determine the risk of potential flooding after snowmelt. Hence this recommendation extends beyond the field of tree phenology but would generally benefit the field of environmental risk assessment under climate change.

## Conclusions

In this study the hypothesis that budbreak timing could be advanced under drier conditions, allowing drought stress to occur earlier was tested. The timing of budbreak was simulated using an eco-physiological model. Then, using four climate models from the CMIP6 a monthly drought index (SPEI) was calculated and the anomaly in the timing of budbreak regressed with the anomaly in the drought index during the month of budbreak. Results showed that drier conditions during the timing of budbreak were more likely at the northern site compared to the southern site. In line with the present hypothesis, drought stress could start earlier in the future. However, it has been shown that the biological inference regarding the timing of budbreak under drier conditions in the future is dependent on the climate model, so this hypothesis requires further testing. Indeed, two climate models expect drier conditions during the timing of budbreak whereas this relation was weakened for two other climate models. Therefore, the use of many climate models is required when assessing tree responses to climate change since climate models can diverge in their outputs. Moreover, if the different phases of precipitation (snow, rain) would be available instead of the daily precipitation sum, it would help in determining the impact rain-on-snow can have on the snow depth accumulation and its potential to reduce water availability in spring. Additionally, the development of a moving drought index would help determine drought exposure at biologically relevant times. Importantly, the methodology presented, which involves the use of many climate models, is critical in determining which sylvicultural treatment and which species to plant and should be used to reduce drought stress in managed forests.

## SUPPLEMENTARY DATA

Supplementary data to this article can be found online.

## Data Availability

The climate data that support the findings of this study are available on the Pacific Climate Impact Consortium (https://data.pacificclimate.org/portal/downscaled_cmip6/map/). The simulated timing of budbreak are already shown in Figs 2 & 3 and the drought index data are shown in Fig. 4.
