# Supplementary material for: Simulations reveal variability in exposure to drier conditions during timing of budbreak for tree species of the mixedwood forests of Québec, Canada
Source: For Res (Fayettev). 2024 Aug 7;4:e026. doi: 10.48130/forres-0024-0023 (PMC11524311; doi:10.48130/forres-0024-0023)
Supplement: Supplementary file 1 — Supplementary data to this article can be found online. [file forres-0024-0023-S1.zip › 10.48130_forres-0024-0023-Suppl-TableS2.pdf]

Table S2. Summary statistics (coefficients, lower (2.5 %), and upper (97.5) values of the confidence interval) of the linear regression analyzing the temporal trends in the drought index (SPEI) per site, year, climate models, and socioeconomic pathways. Bold values highlight statistically significant variable (P-values < 0.05).

Variables	Coefficients	2.50%	97.50%	P-value
**Intercept: Month [January]; Site [North]; SSP [245]**	**-10.05**	**-12.01**	**-8.09**	**2×10^{-16}**
**Year**	**4.96×10^{-3}**	**3.99×10^{-3}**	**5.93×10^{-3}**	**2×10^{-16}**
**Site [South]**	**-1.05**	**-2.10**	**-1.73×10^{-3}**	**0.05**
SSP [585]	0.94	-0.11	1.99	0.08
**Month [October]**	**11.28**	**8.71**	**13.85**	**2×10^{-16}**
Month [November]	0.47	-2.10	3.04	0.72
**Month [December]**	**-2.99**	**-5.56**	**-0.42**	**0.02**
Month [February]	2.22	-0.35	4.79	0.09
**Month [March]**	**4.05**	**1.48**	**6.62**	**2.00×10^{-3}**
**Month [April]**	**9.92**	**7.35**	**12.49**	**3.77×10^{-14}**
**Month [May]**	**16.26**	**13.69**	**18.83**	**2×10^{-16}**
**Month [June]**	**17.99**	**15.42**	**20.56**	**2×10^{-16}**
**Month [July]**	**19.30**	**16.73**	**21.87**	**2×10^{-16}**
**Month [August]**	**17.60**	**15.03**	**20.17**	**2×10^{-16}**
**Month [September]**	**15.16**	**12.59**	**17.73**	**2×10^{-16}**
**Year × Site [South]**	**5.19×10^{-4}**	**1.36×10^{-6}**	**1.04×10^{-3}**	**0.05**
Year × SSP [585]	-50.60	-9.83×10^{-4}	5.19×10^{-5}	0.08
**Year × Month [October]**	**-58.70**	**-6.84×10^{-3}**	**-4.30×10^{-3}**	**2×10^{-16}**
Year × Month [November]	-27.10	1.49×10^{-3}	1.04×10^{-3}	0.72
**Year × Month [December]**	**1.48×10^{-3}**	**2.08×10^{-4}**	**2.74×10^{-3}**	**0.02**
Year × Month [February]	-14	-2.37×10^{-3}	1.72×10^{-4}	0.09
**Year × Month [March]**	**-23**	**-3.27×10^{-3}**	**-7.33×10^{-4}**	**2.00×10^{-3}**
**Year × Month [April]**	**-52**	**-6.17×10^{-3}**	**-3.63×10^{-3}**	**3.68×10^{-14}**
**Year × Month [May]**	**-83.3**	**-9.30×10^{-3}**	**-6.76×10^{-3}**	**2×10^{-16}**
**Year × Month [June]**	**-91.9**	**-1.02×10^{-2}**	**-7.62×10^{-3}**	**2×10^{-16}**
**Year × Month [July]**	**-98.3**	**-1.08×10^{-2}**	**$-8,26 \times 10^{-3}$**	**2×10^{-16}**
**Year × Month [August]**	**-89.9**	**-9.96×10^{-3}**	**-7.42×10^{-3}**	**2×10^{-16}**
**Year × Month [September]**	**-77.9**	**-8.75×10^{-3}**	**-6.21×10^{-3}**	**2×10^{-16}**
