# Supplementary material for: Simulations reveal variability in exposure to drier conditions during timing of budbreak for tree species of the mixedwood forests of Québec, Canada
Source: For Res (Fayettev). 2024 Aug 7;4:e026. doi: 10.48130/forres-0024-0023 (PMC11524311; doi:10.48130/forres-0024-0023)
Supplement: Supplementary file 1 — Supplementary data to this article can be found online. [file forres-0024-0023-S1.zip › 10.48130_forres-0024-0023-Suppl-TableS3.pdf]

Table S3. Summary statistics (coefficients, confidence intervals and P-value) of the linear regression analysis of the anomaly in timing of budbreak predicted by the anomaly in the drought index during timing of budbreak per site, climate model, and socioeconomic pathways.

**Variable**	**Coefficients**	**Confidence interval**		**P-value**
		**Low (2.5 %)**	**High (97.5 %)**	
**Intercept**	**-283.30**	**-309.47**	**-257.09**	**$< 2.00 \times 10^{-16}$**
Anomaly in drought index	0.17	-0.29	0.64	0.46
**Year**	**0.14**	**0.13**	**0.16**	**$< 2.00 \times 10^{-16}$**
**Site [South]**	**-75.95**	**-97.20**	**-54.71**	**2.71×10^{-12}**
**Model [CanESM5]**	**-156.40**	**-186.52**	**-126.21**	**$< 2.00 \times 10^{-16}$**
**Model [GFDL_ESM4]**	**82.52**	**52.33**	**112.71**	**8.78×10^{-8}**
**Model [MIROC6]**	**-52.59**	**-82.98**	**-22.19**	**7.01×10^{-4}**
**SSP [585]**	**-174.70**	**-195.95**	**-153.51**	**$< 2.00 \times 10^{-16}$**
Anomaly_SPEI × Model [CanESM5]	0.36	-0.29	1.01	0.27
**Anomaly_SPEI × Model [GFDL_ESM4]**	**0.90**	**0.24**	**1.55**	**7.08×10^{-3}**
**Anomaly_SPEI × Model [MIROC6]**	**1.36**	**0.71**	**2.02**	**4.66×10^{-5}**
**Year × Site [South]**	**0.04**	**0.03**	**0.05**	**3.39×10^{-13}**
**Year × Model [CanESM5]**	**0.08**	**0.06**	**0.09**	**$< 2.00 \times 10^{-16}$**
**Year × Model [GFDL_ESM4]**	**-0.04**	**-0.06**	**-0.03**	**3.69×10^{-8}**
**Year × Model [MIROC6]**	**0.03**	**0.01**	**0.04**	**4.32×10^{-4}**
**Year × SSP [585]**	**0.09**	**0.08**	**0.10**	**$< 2.00 \times 10^{-16}$**
**Site [South] × Model [CanESM5]**	**-2.00**	**-3.29**	**-0.70**	**2.46×10^{-3}**
**Site [South] × Model [GFDL_ESM4]**	**-2.40**	**-3.69**	**-1.11**	**2.78×10^{-4}**
**Site [South] × Model [MIROC6]**	**-2.82**	**-4.11**	**-1.53**	**1.86×10^{-5}**
**Model [CanESM5] × SSP [585]**	**2.66**	**1.37**	**3.95**	**5.46×10^{-5}**
Model [GFDL_ESM4] × SSP [585]	0.40	-0.89	1.69	0.55
**Model [MIROC6] × SSP [585]**	**1.84**	**0.54**	**3.13**	**5.33×10^{-3}**
