# Supplementary material for: Simulations reveal variability in exposure to drier conditions during timing of budbreak for tree species of the mixedwood forests of Québec, Canada
Source: For Res (Fayettev). 2024 Aug 7;4:e026. doi: 10.48130/forres-0024-0023 (PMC11524311; doi:10.48130/forres-0024-0023)
Supplement: Supplementary file 1 — Supplementary data to this article can be found online. [file forres-0024-0023-S1.zip › 10.48130_forres-0024-0023-Suppl-TableS1.pdf]

Table S1. Summary statistics (coefficients, lower (2.5 %), and upper (97.5) values of the confidence interval) of the linear regression model analyzing temporal trends in timing of budbreak per climate models, socioeconomic pathway, sites, early and late bursting species according to linear regression model. Bold values highlight statistically significant variable (P-values < 0.05).

**Variables**	**Coefficients**	**Confidence interval**	
		**2.50%**	**97.50%**
**Intercept: Site [North]; Early busting species; Model [ACCESS-CM2]; SSP [245]**	**432.38**	**406.23**	**458.52**
**Site [South]**	**61.45**	**40.1**	**82.79**
**Late bursting species**	**15.94**	**15.48**	**16.4**
**Model [CanESM5]**	**157.07**	**126.88**	**187.26**
**Model [GFDL_ESM4]**	**-79.79**	**-109.98**	**-49.61**
**Model [MIROC6]**	**67.52**	**37.33**	**97.71**
**SSP [585]**	**175.92**	**154.56**	**197.28**
**Year**	**-0.14**	**-0.16**	**-0.13**
**Site [South] × Year**	**-0.04**	**-0.05**	**-0.03**
**Model [CanESM5] × SSP [585]**	**-2.65**	**-3.95**	**-1.35**
Model [GFDL-ESM4] × SSP [585]	-0.38	-1.68	0.92
**Model [MIROC6] × SSP [585]**	**-1.84**	**-3.14**	**-0.54**
**Model [CanESM5] × Year**	**-0.08**	**-0.09**	**-0.06**
**Model [GFDL-ESM4] × Year**	**0.04**	**0.03**	**0.06**
**Model [MIROC6] × Year**	**-0.03**	**-0.05**	**-0.02**
**SSP [585] × Year**	**-0.09**	**-0.1**	**-0.08**
